# Supplementary material for: Effect of Increased Depressive Feelings during the COVID-19 Pandemic on the Association between Depressive Mood and Suicidal Behavior: Using the 17th (2021) Korea Youth Risk Behavior Web-Based Survey (KYRBS)
Source: Int J Environ Res Public Health. 2022 Nov 11;19(22):14868. doi: 10.3390/ijerph192214868 (PMC9690650; doi:10.3390/ijerph192214868)
Supplement: Supplementary file 1 [file ijerph-19-14868-s001.zip › ijerph-1959429-supplementary.pdf]

Supplemental Material 1. Checklist for Reporting Results of Internet E-Surveys (CHERRIES)*

Checklist Item	***Explanation***	***Page Number***
Describe survey design	The target population for the survey is nationally representative middle- and high-school students aged 12–18 years in Korea at April 2021. 59426 students from 400 sampled middle and 400 sampled high schools have participated in the survey conducted between august and november in 2021. KYRBS use a multi-stage cluster sampling design to obtain a nationally representative sample of korean students. The target population comprises all public and private middle- and high-school students of 17 provinces in Korea. . In the first stage of the sampling, schools are selected as primary sampling units (PSUs). Within a PSU, one classroom of each grade is sampled using the systematic sampling method.	Page 1 (reference 2)
IRB approval	:This is a secondary data analysis, which is approved by the Institutional Review Board (IRB) of Wonju Severance Christian Hospital (IRB No. CR322330).	Page 9
Informed consent	All participants had already provided informed consent to participate in online surveys. A trained teacher explains the purpose and method of the survey to sampled students using an online video in a computer lab of a sampled school. After that Each sampled student accesses to the website of KYRBS. and check online informed consent.	Page 4 (reference 2)
Data protection	All users must complete the raw data user consent. for the consent of the raw data user, the name, affiliation, and e-mail must be written.	Page 5 (reference 2)
Development and testing	To ensure data quality, survey administrators use standardized operational procedures. Before conducting a survey, Centers for Disease Control and Prevention(CDC) performs nationwide training programmes for teachers (mostly nurse teachers in schools, excluding home teachers of the sampled class) from the sampled schools between April and May each year, to help ensure the standardized data collection of the survey. A trained teacher in each school registered information on the characteristics of schools and the numbers of male and female students of all classes in her/his school via the website of KYRBS [http:// yhs.cdc.go.kr]. This information is used to select classes (one class per each grade) and to create sample weights.	Page 4 (reference 2)
Open survey versus closed survey	KYRBS is a closed survey. each student in sampled schools logs in using the certificate number printed on the information sheet and check online informed consent.	Page 4 (reference 2)
Contact mode	all participants cannot log in the survey before they receive the certificate number printed on the informed sheet. and All participants receive a questionnaire on the day of the survey.	Page 4 (reference 2)
Advertising the survey	KYRBS conducted surveys only to students sampled by the Korea Centers for Disease Control and Prevention and was not advertised.	Page 4 (reference 2)
Web/E-mail	This was a web-based survey.	Page 1

		(reference 2)
Context	KYRBS was posted on the government web site(CDC). However, participants are selected nationwide through a sampling process.	Page 4 (reference 2)
Mandatory/voluntary	KYRBS was a voluntary survey. Unwanted participants do not participate in the survey.	Page 4 (reference 2)
Incentives	A mask was provided as a small reward.	confirmed with the official
Time/Date	the 17th KYRBS was conducted between august and november in 2021.	confirmed with the official
Randomization of items or questionnaires	No randomization of items was used.	Page 1 (reference 2)
Adaptive questioning	Adaptive questioning(branched) was used. Relevant survey items were displayed based on previous responses.(e.g. Only the participants who responded that they had smoking experience saw questions about the frequency of smoking.) Certain items were also populated based on previous responses.	confirmed with the official
Number of Items	A maximum of three items were displayed on any one survey page. The full survey comprised a total of 112 items, although because of the adaptive nature of the questionnaire, not all respondents answered all items.	confirmed with the official
Number of screens (pages)	The full survey was distributed over approximately 95 pages.	confirmed with the official
Completeness check	All survey items were deemed to be mandatory, and respondents prompted to complete outstanding items before leaving the survey page on which the item was contained.	confirmed with the official
Review step	Respondents were unable to change their responses once submitted. If participants want to change their answers, they should ask their health teacher to correct them after the survey.	confirmed with the official
Unique site visitor	all participants received the certificate number and this number identifies the participants.	Page 4 (reference 2)
View rate (Ratio of unique survey visitors/unique site visitors)	Not applicable; respondents were invited through an external panel.	confirmed with the official
Participation rate (Ratio of unique visitors who agreed	A total of 54848 students participated in a total of 59066 subjects, and the participation rate of the survey based on the number of students was 92.9%.	confirmed with the official

to participate/unique first survey page visitors)		
Completion rate (Ratio of users who finished the survey/users who agreed to participate)	Of the 54848 participants who commenced the survey, 54848 completed it, giving a completion rate of 100.00%	confirmed with the official
Cookies used	No	confirmed with the official
IP check	NO, KYRBS use certificate number.	Page 4 (reference 2)
Log file analysis	Not used	Not reported
Registration	Entry to the survey was via a unique certificate number provided to each invitee to the survey.	Page 4 (reference 2)
Handling of incomplete questionnaires	questionnaires that terminated early were also used. Instead data that terminated early were excluded from the study.	confirmed with the official
Questionnaires submitted with an atypical timestamp	The average completion time of the survey was at approximately 15minutes~20minutes. The minimum completed survey was timed at approximately 10 minutes. The average completion time of the survey was at approximately 15minutes~20minutes. No respondents were removed from the survey for completing the items too quickly.	confirmed with the official
Statistical correction	KYRBS is provided after the process of weight generation and stratification.<br>The weight is the value obtained by multiplying the reciprocal of the extraction rate and the inverse of the response rate by the post-weighting correction rate.	confirmed with the official

* Eysenbach G. Improving the quality of Web surveys: the Checklist for Reporting Results of Internet E-Surveys (CHERRIES). J Med Internet

Supplemental Material 2. Health Behavioral characteristics Questions

Variables	Questions	Answers
Grade	What grade are you in?	① 10th<br>② 11th<br>③ 12th
Academic performance	In the last 12 months, how is your academic performance?	① High<br>② Medium high<br>③ Medium<br>④ Medium low<br>⑤ Low
Economic status and deterioration post-COVID 19	What is the economic status of your family?	① High<br>② Medium high<br>③ Medium<br>④ Medium low<br>⑤ Low
	Do you think the economic condition of student families is more difficult than before due to COVID-19?	① Strongly agree<br>② Agree<br>③ Disagree<br>④ Strongly disagree
Type of residence	What is your current residence?	① Live with one's family<br>② Live in a relative's house<br>③ Boarding house, living alone<br>④ Dormitory<br>⑤ Childcare facilities (Orphanages, Social welfare

		facilities, Nurseries)
Post-COVID 19 physical activity	Before the COVID-19 pandemic, is there any change in comparison? (in physical activity)	① Increased very much<br>② Increased<br>③ No change<br>④ Decreased<br>⑤ Very reduced
Tobacco use	In the last 30 days, how many days have you smoked a regular cigarette?	① None in the last 30 days<br>② 1~2 days per month<br>③ 3~5 days per month<br>④ 6~9 days per month<br>⑤ 10~19 days per month<br>⑥ 20~29 days per month<br>⑦ Every day
	In the last 30 days, how many days did you use liquid e-cigarettes containing nicotine?	① None in the last 30 days<br>② 1~2 days per month<br>③ 3~5 days per month<br>④ 6~9 days per month<br>⑤ 10~19 days per month<br>⑥ 20~29 days per month<br>⑦ Every day
	In the last 30 days, how many days did you use cigarette-type e-cigarettes (heated cigarettes, e.g., IQOS, Glo, Lil, etc.)?	① None in the last 30 days<br>② 1~2 days per month<br>③ 3~5 days per month<br>④ 6~9 days per month<br>⑤ 10~19 days per month

		⑥ 20~29 days per month<br>⑦ Every day
Alcohol use	In the last 30 days, how many days did you have at least 1 drink?	① None in the last 30 days<br>② 1~2 days per month<br>③ 3~5 days per month<br>④ 6~9 days per month<br>⑤ 10~19 days per month<br>⑥ 20~29 days per month<br>⑦ Every day
Perceptual body image	What do you think of your body shape?	① Skinny<br>② Slightly skinny<br>③ Normal<br>④ Slightly fat<br>⑤ Fat
Sleep satisfaction	In the past 7 days, do you think the amount of sleep you have slept is sufficient to recover from fatigue?	① Very good enough<br>② Enough<br>③ Just so<br>④ Not enough<br>⑤ Not enough at all
